# A Novel Inducible mtDNA Mutator Mouse Model to Study Mitochondrial Dysfunction with Temporal and Spatial Control

**DOI:** 10.1093/geroni/igaf122.534

**Published:** 2025-12-31

**Authors:** Hannah Tobias-Wallingford, Sydney Bartman, Lauren Gaspar, Giuseppe Coppotelli, Jaime Ross

**Affiliations:** University of Rhode Island, Kingston, Rhode Island, United States; University of Rhode Island, Kingston, Rhode Island, United States; University of Rhode Island, Kingston, Rhode Island, United States; University of Rhode Island, Kingston, Rhode Island, United States; University of Rhode Island, Kingston, Rhode Island, United States

## Abstract

Mitochondrial dysfunction is a hallmark of aging and numerous age-related diseases. A wealth of studies supports the accumulation of mitochondrial DNA (mtDNA) mutations as a driving factor of mitochondrial dysfunction in aging and disease. One of the best models to study the relationship between mtDNA mutations and mitochondrial dysfunction is the mtDNA mutator mouse, which expresses a proofreading-deficient version of mtDNA polymerase-gamma (PolgA), resulting in accelerated accumulation of mtDNA mutations and aging-like mitochondrial dysfunction. Despite its many contributions in mitochondrial biology and aging research, this model is limited by the whole-body accumulation of mtDNA mutations, which prevents the investigation of tissue-specific differences in mitochondrial dysfunction. To overcome this limitation, we developed a novel inducible knock-in mtDNA mutator mouse model that allows spatial and temporal control of mtDNA mutations, enabling the precise study of mitochondrial dysfunction in a tissue- and time-specific manner. Here, we report the generation and validation of this novel model through whole-body induction via Cre recombinase. Our data demonstrate that, upon induction, this model recapitulates the phenotype of the original mtDNA mutator mouse manifesting the same behavioral and biochemical alterations. This work establishes the functionality of our model and highlights its value as a powerful tool for studying the impact of mtDNA mutations on aging and age-related disorders with enhanced specificity and control.

